# On-site urine treatment combining Ca(OH)_2_ dissolution and dehydration with ambient air

**DOI:** 10.1016/j.wroa.2021.100124

**Published:** 2021-10-08

**Authors:** Michel E. Riechmann, Bonginkosi Ndwandwe, Esther E. Greenwood, Eva Reynaert, Eberhard Morgenroth, Kai M. Udert

**Affiliations:** a Eawag, Swiss Federal Institute of Aquatic Science and Technology, 8600 Dübendorf, Switzerland; b ETH Zürich, Institute of Environmental Engineering, 8093 Zürich, Switzerland; c University of KwaZulu Natal, WASH R&D Center, 4041 Durban, South Africa

**Keywords:** Field testing, Resource recovery, Urine stabilization, Calcium hydroxide, Source separation, Blue Diversion Autarky

## Abstract

We present the results of three field tests and three laboratory tests of a new physical-chemical urine treatment system, which can recover all nutrients, while pathogens are inactivated. The system consists of two steps. In the first reactor, biological processes including urea hydrolysis are prevented by mixing fresh urine with calcium hydroxide (Ca(OH)_2_). Due to the high pH value and the high availability of calcium, phosphate can be recovered by precipitation. The high pH value also fosters the inactivation of microorganisms, including pathogens. In the second reactor, water is evaporated at low energy consumption by blowing unheated ambient air over the stabilized urine. Stabilization in the first reactor was successful in all field and laboratory tests. The pH value remained between 12 and 13, except for short dips due to shortages of Ca(OH)_2_. Nearly all phosphorus (92-96%) precipitated and could be recovered as calcium phosphate in the first reactor, while nitrogen and potassium overflowed with the urine into the evaporation reactor. The efficiency of the second treatment step was very different for field and laboratory experiments and depended on the duration of the experiment. During a four-day laboratory test, nitrogen recovery was 98%. In contrast, nitrogen recovery was only around 20% in the long-term field experiments. The high nitrogen losses occurred, because biological urea hydrolysis was not inhibited anymore, when the pH value in the second reactor decreased due to the dissolution of high amounts of carbon dioxide from the ambient air. Potassium was not subject to any significant loss, and the measured recovery in the solid evaporation product was 98%. Evaporation rates ranged between 50 g m^−2^ h^−1^ (RH = 82±13%, T = 12±6°C) and 130 g m^−2^ h^−1^ (RH = 60±19%, T = 24±5°C) in the three field tests. Apart from some disturbances due to low supply of Ca(OH)_2_, the urine module functioned without any substantial failures and was simple to maintain. The minimum consumption of Ca(OH)_2_ at full capacity was 6 g·L^−1^ urine and the electricity demand was 150 Wh kg^−1^ water evaporated from urine, resulting in operational costs of 0.05 EUR pers^−1^ d^−1^.

## Highlights

• Two field tests in Switzerland and one in South Africa.
• Short-term stabilization and evaporation resulted in 98% nitrogen recovery.
• During long-term field tests nitrogen recovery decreased to 20%.
• Evaporation rates were governed by the relative humidity of the ambient air.
• Low resource consumption resulted in costs as low as 0.05 EUR pers^−1^ d^−1^.

## Graphical abstract

**Figure:**
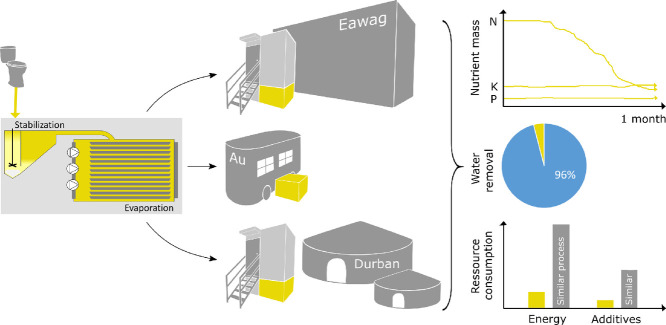

## 1 Introduction

Over the past years, non-sewered sanitation systems have received increasing attention as an alternative approach to centralized sewer-based wastewater treatment, especially for countries with no or only deficient sanitation infrastructure. One of the concepts used for non-sewered sanitation systems is the separate collection and treatment of urine, feces and greywater (Larsen et al. 2013). This concept allows tailoring the treatment according to the specific properties of the three waste streams. The main goal of urine treatment is the recovery of nutrients, since urine contains approximately 80% of nitrogen, 50% of phosphorus and 70% of potassium found in typical wastewater (Friedler et al. 2013). When treating urine, stabilization is a necessary first step to prevent nitrogen loss due to ammonia volatilization after urea hydrolysis (Siegrist et al. 2013) and malodor resulting from fermentation (Troccaz et al. 2013). When urine is stabilized, water can be safely removed for example by evaporation with forced convection (Bethune et al. 2014).

A strong pH increase in fresh urine through base dosage is one option for urine stabilization particularly well suited for onsite urine treatment (Larsen et al. 2021). Randall et al. (2016) described alkaline stabilization by dosing calcium hydroxide (Ca(OH)_2_). By mixing urine with Ca(OH)_2_, the pH increases to values of 12.5 at which the solubility equilibrium for Ca(OH)_2_ is reached. At this high pH, biological urea hydrolysis is inhibited, phosphate precipitates as amorphous calcium phosphate (Randall et al. 2016) and most pathogens are quickly inactivated (Senecal et al. 2018). However, urine stabilized with base is sensitive to high temperatures and pH changes. Randall et al. (2016) recommend to keep the temperature below 40°C and to maintain pH values below 13 to prevent chemical urea hydrolysis. Furthermore, they recommend a minimum pH value of 11 to suppress biological urea hydrolysis. Around 5% of the initial nitrogen leave the body as ammonia and can thus not be stabilized with this method (Udert et al. 2006).

The first study of a urine treatment system combing alkaline urine stabilization and water evaporation with forced convection was reported by Dutta and Vinnerås (2016). The two processes, stabilization and water removal were combined in one reactor and a 1:1 mixture of wood ash and Ca(OH)_2_ was used as alkaline medium. Since water removal was very low at ambient air temperatures, the air was heated up to 60°C, which led to a diminished nitrogen retention of only 46%. The process was optimized by the same research group using various alkaline calcium- and magnesium-containing materials, such as wood ash (Senecal and Vinnerås 2017), Ca(OH)_2_ (Senecal and Vinnerås 2017, Simha et al. 2020b) and magnesium hydroxide (Vasiljev et al. 2022). Simha et al. (2020b) could show that independently of the alkaline medium, more than 90% of the nitrogen could be recovered, if the pH value in urine was kept above 12. They could also show that the recovery was higher with air at 60°C than at 50°C, because the faster evaporation rate at 60°C reduced the duration of the experiment. However, in a field experiment with a similar reactor setup (Simha et al. 2020a) only 30% of the nitrogen was recovered and the electricity demand was high (24.5 kWh kg_urine_^−1^). The high specific electricity demand was caused by the use of heating fans and an unexpected low urine load.

In this study, we tested the urine treatment module of the Blue Diversion Autarky Toilet (BDAT, www.autarky.ch). The goal of the BDAT project was to develop a non-sewered sanitation system for 10 users, which can provide safe sanitation, comfort and resource recovery while operating completely independently of the grid. The urine treatment module of the BDAT is also based on alkaline stabilization and evaporation by forced convection. However, there are some substantial differences to the previously reported systems: urine stabilization and water evaporation are separated in two sequential reactors, the air is not heated and the driving force is instead enhanced through a high air flow. This combines with a larger specific surface area in the evaporation reactor, i.e. 125 m^2^ m^−3^ surface area to volume ratio of the urine compared to 3 m^2^ m^−3^ as in the field tests of Simha et al. (2020a). Furthermore, we only used pure Ca(OH)_2_ as base without blending with other alkaline substances. We chose this setup with the following intentions: phosphate should be recovered as a separate high value fertilizer product in the stabilization reactor, the larger specific area in the evaporation reactor should allow for a high evaporation rate without the need for air heating, and separating stabilization and evaporation should minimize Ca(OH)_2_ consumption. Simha et al. (2018) discussed that CO_2_ absorption during evaporation could induce CaCO_3_ precipitation, which would lead to an increased Ca(OH)_2_ consumption. Finally, alkaline stabilization and subsequent water removal was barely tested at field scale, which is why we included extensive field testing in different locations and setups. We expected two major challenges for our setup. First, limited predictability of evaporation as it strongly depends on climatic conditions. Second, CO_2_ absorption in the evaporation reactor could decrease the pH, so that biological urea hydrolysis would become possible. However, it was shown that urease, the enzyme responsible for biological urea hydrolysis, is increasingly inhibited at a rising ionic strength (Hotta and Funamizu 2008, Kumar and Kayastha 2010). We therefore presumed that the increase of salt concentrations during evaporation would prevent biological urea hydrolysis, even if the pH decreases below 11.

The overall aim of this study was to test whether the urine module of the BDAT was suited to concentrate all urine nutrients of a 10 people household. We conducted basic laboratory tests for a proof of concept, controlled short-term tests with the urine treatment module and three field tests, two in Switzerland and one in South Africa. The overall study was guided by three basic research questions:

i Can urea degradation and subsequent nitrogen loss be prevented?
ii Is the evaporation rate sufficiently high to remove the water from the collected urine?
iii Are the operational costs reasonably low?

Additionally we determined the mass flows of nitrogen, phosphorus and potassium.

## 2 Materials and Methods

### 2.1 The urine module in the Blue Diversion Autarky Toilet

**Fig. 1:**
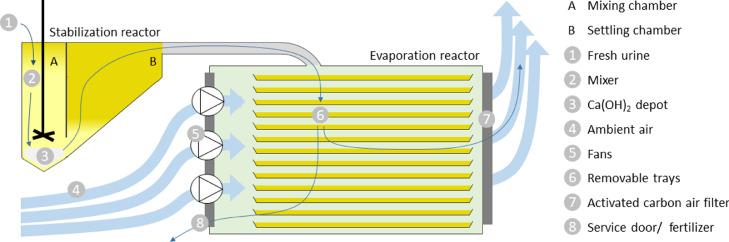
Schematic diagram of the BDAT urine module. Fresh urine enters the stabilization reactor (1). The stabilized urine overflows to the evaporation reactor (6). Ambient air (4) flows through the stacked tray system to force evaporation of water and produce a concentrated product.

The urine module of the BDAT is designed for a 10 people household or an equivalent of 10 L urine per day and 3 L flushing water per day (Table 1). The reactor setup consists of two treatment units: urine stabilization and water evaporation (Fig. 1). Urine stabilization is achieved by increasing the pH value through the dissolution of Ca(OH)_2_. Due to its limited solubility, just enough Ca(OH)_2_ dissolves to maintain a pH value of about 12.5. The remaining Ca(OH)_2_ is available for future urine charges. A Ca(OH)_2_ amount sufficient to operate the urine module for several weeks is added during each service. The stabilization reactor has a volume of 25 L and is divided into a mixing and a settling chamber. The urine is mixed every hour for three minutes at 340 rpm. In the settling chamber, unused Ca(OH)_2_ and precipitates, mainly calcium and magnesium phosphate minerals, are separated from the liquid and slide back into the mixing chamber. To avoid CaCO_3_ precipitation following CO_2_ absorption from the air, the stabilization reactor is sealed against incoming air with a membrane valve.

In the second treatment unit, water is evaporated from the overflown liquid by forced air convection. 12 stainless steel trays (LxW: 930 × 560 mm^2^) provide a total evaporation area of 6.2 m^2^. Urine is distributed to a lower tray when the water level exceeds a natural overflow of 8 mm, which is integrated at the front of the trays. Nine axial fans (G1238 E 24 B1+6, Mechatronics Inc., Preston, USA) continuously blow ambient air over the trays during operation of the evaporation reactor. In all long-term field tests, the evaporation reactor was operated for 10 h during daytime, in order to take advantage of the lower RH during daytime. To remove malodorous volatile organic gases, a 1 cm thick activated carbon filter mat can be inserted at the outflow of the tray stack. When integrated in the BDAT, the outgoing air is discharged over the roof of the toilet cabin. During service, excess urine can be drained by inclining the trays and the remaining solid fraction can be harvested with a scraper. To test different influences (e.g. on the energy demand) the air filter at the back of the evaporation reactor was removed during selected periods.

### 2.2 Long-term field tests

The urine module was tested in three different field settings. One field test was conducted on Eawag campus (FT_Eawag_) with an automatic feed of fresh urine in order to control and measure the input of the urine module. The two field tests in Au (FT_Au_: Zurich/Switzerland) and Durban (FT_Durban_: South Africa), were carried out to evaluate the performance and adaptability of the module in different real-world contexts. A socio-technical evaluation of the field test of the BDAT (including the urine module) in Durban is described in Sutherland et al. (2020). Table 1 provides further background information about the field experiments (photographs and detailed setup in Figure S.1 to S.4 of the Supplementary Information).

**Table 1:** Testing conditions and reactor specifications of the long-term field tests FT_Eawag_, FT_Au_ and FT_Durban_.

	FT_Eawag_	FT_Au_	FT_Durban_
Location	Eawag campus, Dübendorf, Switzerland	Peri-urban zone of Au (Zürich), Switzerland	Unsewered peri-urban zone of Durban, South Africa
Setup	Urine module test	Urine module test	Blue Diversion Autarky Toilet
Research focus	Mass balances of water and nutrients	pH-indicated Ca(OH)_2_ dosing	Operation in a challenging environment
Urine collection	External urine collection at Eawag office building, storage at 4°C until use	Direct connection to urine diverting dry toilet (UDDT)^1^	Direct connection to waterless urinal^2^ and water-flushed urine diverting toilet^3^
Input feed	4.3 L·d^−1^ fresh urine (pH <7, weekdays) and pre-stabilized urine (6 g_Ca(OH)2_·L^−1^, weekends), both diluted with tap water^4^ (75:25)	Undiluted fresh urine of 2 people	Fresh urine of 14 people diluted with toilet flush water
Ca(OH)_2_ dosing^5^	800 g through weekly dosing of irregular amounts	180 g dosed if pH <11.5	1800 g at start of each harvest cycle
Duration	28 days (one harvest cycle); August 2019 (late summer)	112 days (one harvest cycle); August to November 2019 (late summer to early winter)	93 days (two harvest cycles of 43 and 50 days); April to June 2019 (autumn)
Climatic conditions^6^	Relative humidity (RH): 28-94% (60%) Temperature: 15-36°C (24°C)	41-98% (82%) 0-31°C (12°C)	21-96% (77%) 7-34°C (21°C)
Sampling	Regularly (2-3 times/week): fresh and stabilized urine End: (Triplicate grab samples): Stabilization reactor effluent, excess urine and urine concentrate from trays	Stabilized urine Stabilization reactor effluent and mixed urine concentrate from trays	Stabilized urine Stabilization reactor effluent, Excess urine and urine concentrate from trays
Measurements	Constantly: pH in mixer, settler and evaporator (manually), inflow temperature and RH, inflow masses End: Product masses	pH in mixer, settler and evaporator (manually), inflow temperature and RH Product masses	pH in mixer, settler and evaporator (manually), inflow temperature and RH, air flow, Ammonia in off-gas Product masses

1 Villa 9000, Separett, Värnamo, Sweden.

2 Lema, Laufen Bathrooms, Laufen, Switzerland.

3 Save! prototype 4, EOOS, Vienna, Austria.

4 Simulating toilet flush water entering the system.

5 Ca(OH)_2_, technical grade (≥95%), VWR Chemicals, Darmstadt, Germany.

6 Ranges with average values in parenthesis during reactor operation hours. For detailed climate conditions see Table S.1 and Figure S.5.

To reduce the amount of urine needed for FT_Eawag_, the input volume was reduced. The daily inflow of 4.3 L diluted urine corresponded to one third of the design load. Table 2 gives an overview of the composition of the urine used in the different field tests.

**Table 2:** Characterization of incoming urine in the field tests given as mean ± standard deviation. In FT_Eawag_ it was possible to test the fresh urine inflow batches, while in the case of FT_Au_ and FT_Durban_ the sampling took place in the stabilization reactor. For FT_Durban_, fresh urine was also collected directly from the users during one day to determine the concentrations in the input urine. Parameters not determined are left blank.

Parameter	FT_Eawag_	FT_Au_	FT_Durban_	FT_Durban_
Type	Diluted fresh urine (75:25)^2^	Undiluted stabilized urine^3^	Undiluted fresh urine	Diluted stabilized urine (30:70)^2^^,^^3^
Number samples n^1^	12	4	Triplicates	11
Total organic carbon [mg·L^−1^]	4270±870 (n=11)	5860±400	7710±130	
Chemical oxygen demand [mg·L^−1^]				2690±270 (n=3)
Total nitrogen [mg_N_·L^−1^]	4600±1280	6000±470	9250±80	2080±180
Urea [mg_N_·L^−1^]	3090±820	4320±410	7640±110	
Total ammonia [mg_N_·L^−1^]	143±31	761±146	501±6	160±50
Nitrate [mg_N_·L^−1^]				23±4 (n=2)
Nitrite [mg_N_·L^−1^]				6±2 (n=6)
Total phosphorus [mg_P_·L^−1^]			700±14	8±11 (n=8)
Orthophosphate [mg_P_·L^−1^]	195±65	15±11	660±9	
Potassium [mg·L^−1^]	1320±390	1560±150	1360±20	510±70
Sulphate [mg·L^−1^]	508±114	1330±170	1740±50	
Chloride [mg·L^−1^]	2330±280	3570±220	4690±70	1360±120
Sodium [mg·L^−1^]	1140±180	1990±110	3360±30	940±80
Calcium [mg·L^−1^]	90±55 (n=10)		71±9	640±480 (n=4)
Magnesium [mg·L^−1^]	73±25		90±4	58±36 (n=6)
pH	6.5±0.4	12.2±0.4	5.9	12.4±0.2

1 Deviating sample numbers in brackets due to test kit shortages or invalid measurements.

2 Dilution ratio urine to tap water.

3 Stabilized with Ca(OH)_2_.

### 2.3 Short-term experiments

Three short-term batch experiments were conducted for in-depth studies of the reactor performance. In experiments Stab_Lab_ (simplified laboratory setup) and Stab_Field_ (urine module), we focused on the nitrogen loss due to urea hydrolysis during evaporation. In experiment Evap_Field_ (urine module), we examined the effects of the relative humidity (RH) and temperature of the incoming air on the evaporation rate. Details of all three experiments are summarized in Table 3.

**Table 3:** Description of short-term batch experiments at laboratory scale (Stab_Lab_) and using the BDAT urine module under controlled conditions (Stab_Field_ and Evap_Field_)

	Stab_Lab_	Stab_Field_	Evap_Field_
Setup	Laboratory basins with fans placed above - continuous ventilation for urine dehydration	Urine module – continuous ventilation for urine dehydration	Urine module – continuous ventilation for urine dehydration
Research focus	Urea stability during dehydration at laboratory conditions	Urea stability during dehydration at field conditions	Maximum evaporation rate
Input feed	(a) Fresh urine (b) Stabilized^1^ urine (excess Ca(OH)_2_ and calcium phosphate precipitates removed by decantation) (c) Stabilized^1^ urine (including excess Ca(OH)_2_ and calcium phosphate precipitates)	(a) Stored urine (b) Stabilized^1^ urine diluted with tap water^2^ (75:25)	Stabilized^1^ urine diluted with tap water^2^ (75:25)
Input amount	(a, b, c) 1 L	(a) 1.9 L per tray (12 trays) (b) 4 L per tray (4 trays)	4 L per tray (4 trays)
Duration	(a, b, c) 4 d	(a) 4 d (b) 3 d	3 replicates of 1-3 d
Measurements^3^	(a, b, c) pH, total nitrogen (N_tot_), chloride (triplicates at start and end)	(a) pH (start); N_tot_, total ammonia (NH_tot_), chloride (single samples, daily) (b) pH (start); N_tot_, NH_tot_, urea, chloride (single samples at start and end)	Reactor weight logged in 1 min intervals; inflow RH and temperature logged in 10 min intervals

1 Stabilized with 10 g_Ca(OH)2_·L^−1^ urine.

2 Simulating flush water entering the system.

3 The measurement devices used for these experiments are the same as used in FT_Eawag_ described in sections 2.4 to 2.6.

### 2.4 Online monitoring

Process stability was measured with pH as a proxy in all field tests. Two pH sensors for pH measurement (Orbisint CPS11D with multichannel transmitter Liquiline CM444, Endress+Hauser, Reinach, Switzerland) were placed in the mixing and in the settling chamber of the stabilization reactor, respectively. The pH on the trays of the evaporation reactor was measured with a portable pH-meter (Multi-340i multimeter with SenTix 41-3 pH sensor, WTW, Weilheim, Germany) or with color-fixed indicator stripes (pH-Fix 0-14, Machery-Nagel, Düren, Germany).

Ambient RH and temperature of the air inflow were measured and logged with an MSR-145 (MSR Electronics, Seuzach, Switzerland) in ten-minute intervals. In FT_Durban_, the air flow (monitoring station MKA-R-DD-160-VRP-M-VFP300, Schako, Givisiez, Switzerland) and ammonia in the off-gas stream (MONOline 514, Kimessa, Zurich, Switzerland) were measured online.

In the experiment Evap_Field_, the weight of the evaporation reactor was continuously determined with a scale (IKT 150K2XL, Kern, Balingen-Frommern, Germany) and the measurement results were logged in one-minute intervals.

### 2.5 Sampling

Regular samples were taken from the stabilization reactors during all field tests. For FT_Eawag_, incoming fresh urine was also sampled and weighed. At the end of a harvest cycle, the systems were emptied to perform mass balances. The excess urine was drained from the trays of the evaporation reactor, then the remaining solid/liquid mixture, i.e. the evaporation product, was scraped of the trays. In addition, the urine and the precipitation sediments from the stabilization reactor were drained. The masses of the three fractions excess urine, solid evaporation product and stabilization drainage effluent were weighted on site (FT_Eawag_: Champ II, OHaus Europe, Nänikon, Switzerland; FT_Au_: IKT 150K2XL, Kern, Balingen-Frommern, Germany; FT_Durban_: CPWplus-35, Adam Equipment, Oxford, USA). Triplicate grab samples were taken from each mixed and homogenized fraction.

### 2.6 Analytical Methods

For FT_Eawag_ and FT_Au_, ortho-phosphate, sulfate, chloride, sodium, potassium, calcium and magnesium were measured with ion chromatography (930 Compact IC-Flex and 881 Compact IC-Pro, Metrohm, Zofingen, Switzerland), total organic carbon and total nitrogen (N_tot_) were measured with a Shimadzu TOC-L (Kyoto, Japan), and a Lachat QC8500 FIA (HACH, Düsseldorf, Germany) was used for total ammonia (NH_tot_) and urea determination. For FT_Durban_, total phosphorus, N_tot_, nitrate, nitrite, NH_tot_, chloride, sodium, potassium, calcium and magnesium were measured with Merck Spectroquant kits (Darmstadt, Germany), E. coli and total coliforms with an enzyme activity test (Colilert-18-Quanti Tray 2000, IDEXX Laboratories, Maine, USA) and helminth eggs by sieving at 100 and 20 µm, centrifugation and examination under the microscope. Details for all methods applied are available in Table S.2.

### 2.7 Calculations

To compare all experiments in terms of nitrogen recovery efficiency, chloride (Cl) was used as a tracer to normalize for the changes in concentration (Equation 1 and Equation 2). Based on previous measurements, we assumed that chloride did not precipitate in the stabilization reactor.

(1) $$R_{\text{Ntot},\text{stab}}=\frac{C_{\text{Ntot},\text{out},\text{stab}}/C_{\text{Cl},\text{out},\text{stab}}}{C_{\text{Ntot},\text{in},\text{stab}}/C_{\text{Cl},\text{in},\text{stab}}}$$

(2) $$R_{\text{Ntot},\text{evap}}=\;\frac{C_{\text{Ntot},\text{out},\text{evap}}/C_{\text{Cl},\text{out},\text{evap}}}{C_{\text{Ntot},\text{in},\text{evap}}/C_{\text{Cl},\text{in},\text{evap}}}$$

Chloride concentrations were also used to calculate the total amount of urine that entered the system in FT_Au_ and FT_Durban_ (Equation 3), because the volume entering the urine module could not be directly measured. This was done with the in- and outflow compositions of the evaporation reactor.

(3) $$V_{\mathrm{urine},\mathrm{in}}=\frac{m_{\mathrm{Cl},\mathrm{out},\mathrm{evap}}}{C_{\mathrm{Cl},\mathrm{in},\mathrm{evap}}}+V_{\mathrm{stab}}$$

Mass balances of the water content and the nutrients nitrogen, phosphorus and potassium over one harvest cycle were carried out for the experiments Stab_Field_ and FT_Eawag_ using Equation 4 to Equation 10. The system boundaries were drawn around the stabilization and evaporation reactors separately.

(4) $$m_{i}=C_{i}\cdot V_{i}$$

(5) $$m_{i,\mathrm{in},\mathrm{stab}}=m_{i,\mathrm{vola},\mathrm{stab}}+m_{i,\mathrm{outC},\;\mathrm{stab}}+m_{i,\mathrm{outE},\;\mathrm{stab}}$$

(6) $$m_{i,\mathrm{outC},\;\mathrm{stab}}=m_{i,\mathrm{in},\mathrm{evap}}$$

(7) $$m_{i,\mathrm{in},\mathrm{evap}}=m_{i,\mathrm{vola},\mathrm{evap}}+m_{i,\mathrm{outE},\mathrm{evap}}$$

(8) $$R_{i,\mathrm{stab}}=\frac{m_{i,\mathrm{outC},\mathrm{stab}}+m_{i,\mathrm{outE},\mathrm{stab}}}{m_{i,\mathrm{in},\;s\text{tab}}}$$

(9) $$R_{i,\text{evap}}=\frac{m_{i,\text{outE},\text{evap}}}{m_{i,\text{in},\;\text{evap}}}$$

(10) $$R_{i,\text{tot}}=\frac{m_{i,\text{outE},\text{stab}}+m_{i,\text{outE},\text{evap}}}{m_{i,\text{in},\;\text{stab}}}$$

With parameters

m: total mass of one experimental cycle

C: concentration

V: total volume of one experimental cycle

R: recovery efficiency and indices

i: N_tot_, total phosphorus, potassium or water

N_tot_: total nitrogen

Cl: chloride

in: total input to one reactor during one experimental cycle

outC: total output that leaves the stabilization reactor continuously during one experimental cycle

outE: total accumulated reactor content at the end of each experimental cycle

vola: volatilized

stab: stabilization reactor

evap: evaporation reactor

## 3 Results and discussion

### 3.1 Nutrient stabilization and recovery

#### 3.1.1 Nitrogen stabilization

**Fig. 2:**
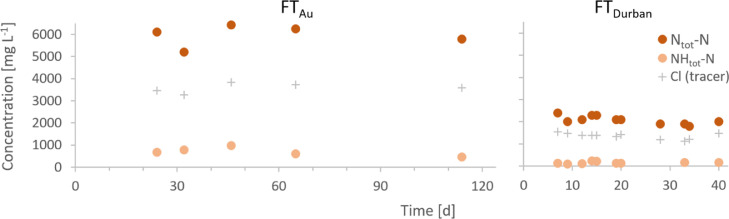
Total nitrogen (N_tot_) and ammonia (NH_tot_) concentrations in the stabilization reactor during the field tests FT_Au_ and FT_Durban_. The tracer chloride (Cl) is given as a reference for inflow concentration variations.

The measurements of NH_tot_ and N_tot_ show that continuous and reliable urea stabilization was achieved inside the stabilization reactor (Fig. 2). The mean NH_tot_:N_tot_ ratios in FT_Eawag_, FT_Au_ and FT_Durban_ were 4%, 7% and 13%, respectively and therefore close to 5%, which is a typical value for fresh urine (Udert et al. 2006). If urea was degraded to a substantial degree, a much larger fraction of N_tot_ would have been present as NH_tot_. Fluctuations of the N_tot_ concentration are probably due to variable inflow concentrations and not to urea degradation or nitrogen volatilization, since the conservative tracer chloride (no degradation and no volatilization during urine stabilization) exhibited a very similar behaviour as N_tot_.

**Fig. 3:**
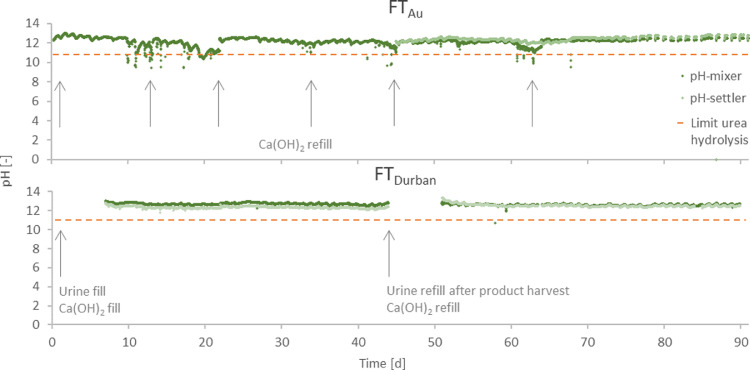
Online monitoring of the stabilization reactor during the field tests FT_Au_ and FT_Durban_. The reactors’ mixing compartment is represented by pH_mixer_ the settling compartment by pH_settler_. The critical limit for enzymatic urea hydrolysis is pH 11. Arrows indicate refilling of Ca(OH)_2_ and urine.

Long-term measurements during the field tests FT_Au_ and FT_Eawag_ revealed that the pH continuously ranged between 12 and 13 with the exception of very few events with lower pH values (Fig. 3). According to a previous study by Randall et al. (2016), pH values between 11 and 13 and temperatures below 40°C are optimal for urea stabilization. At pH values above 11, enzymatic urea hydrolysis is suppressed and significant chemical urea hydrolysis requires pH values above 13 or temperatures above 40°C. High temperatures have been the reason for urea hydrolysis and nitrogen loss in previous studies. In field tests with urine stabilized with base, Simha et al. (2020a) reported nitrogen recoveries of only 30±6% when using 60°C hot air for evaporation. Dutta and Vinnerås (2016) reported nitrogen recoveries of 74% and 54% using temperatures of 35°C and 60°C. However, it seems to be possible to also limit the chemical urea hydrolysis and N_tot_ loss below 10% at temperatures up to 60°C when using high air flow rates and thereby reducing the time while urine is exposed to high temperatures (Simha et al. 2020b).

We tested two different strategies to ensure the required high pH values. The most reliable pH control was achieved in FT_Durban_, when all Ca(OH)_2_ (1.8 kg) predicted to be required for the whole field test was added in the beginning. Due to the high dosage, there was no problem with low pH values during the whole experiment. To increase the dosing efficiency and to reduce the amount of residual Ca(OH)_2_ in the end product, a different dosing regime was tested in FT_Au_, where Ca(OH)_2_ was refilled when the pH value dropped below 11.5. Our experiments showed that short periods with pH values below 11 can occur with the intermittent dosage regime, but they did not cause substantial urea degradation. Between days 10 and 20, the pH values in FT_Au_ showed strong fluctuations. However, the frequent pH drops were most probably due to enzymatic urea hydrolysis in a siphon, installed in front of the stabilization reactor. Longer urine retention times in the siphon, e.g. at night, might have favored enzymatic urea hydrolysis and thereby the release of ammonium and bicarbonate, which act as acids in solutions with high pH values. The strong fluctuation and low pH values did not occur anymore after the siphon was removed on day 23. This incident highlights that the fresh urine must be transported quickly into the stabilization reactor to prevent urea hydrolysis.

#### 3.1.2 Nitrogen recovery

**Fig. 4:**
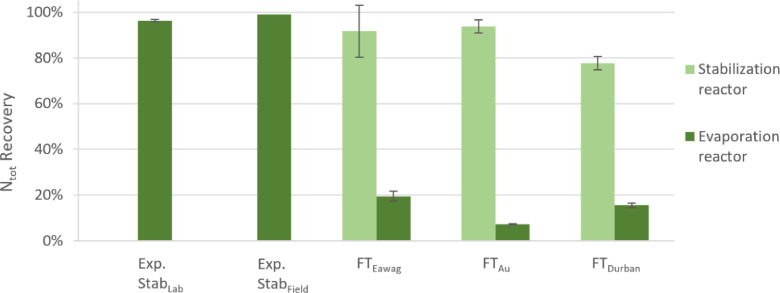
Comparison of N_tot_ recovery efficiencies in experiments Stab_Lab_ and Stab_Field_ and during field tests FT_Eawag_, FT_Au_ and FT_Durban_. Values were obtained by using Equation 1 and Equation 2. For Stab_Lab_ and Stab_Field_ no separate measurements for the stabilization phase were conducted. Error bars indicate the standard deviation measured in triplicate product samples. Only in the cases of the stabilization reactor inflows of FT_Eawag_, FT_Au_ and FT_Durban_ the standard deviation shows the inflow variations over time.

By using Equation 1 and Equation 2, we quantified the recovery efficiencies for nitrogen (Fig. 4). The laboratory results from experiment Stab_Lab_ show that in a period of four days 97±2% of the nitrogen can be recovered using a dehydration process with active ventilation on trays, if urine is stabilized with Ca(OH)_2_ (Figure S.6). A three day short-term run of the urine module under field conditions (Stab_Field_) showed similarly high recovery efficiencies: a comparison of the N_tot_ to chloride ratio at the start and at the end of the experiment showed almost complete N recovery (Figure S.7). Only about the amount of NH_tot_ present before evaporation (130 mg_N_·L^−1^) volatilized during the evaporation of stabilized urine, resulting in a total N loss of 3%. A second batch run of the urine module fed with stored urine (NH_tot,in_=4600 mg_N_·L^−1^) showed that without stabilization, nearly all nitrogen (97%) will be lost: in this experiment, the N_tot_ concentration decreased from an initial concentration of 6300 mg_N_·L^−1^ to 200 mg_N_·L^−1^ within four days (Table S.3). This clearly shows the importance of stabilization for nitrogen recovery.

**Fig. 5:**
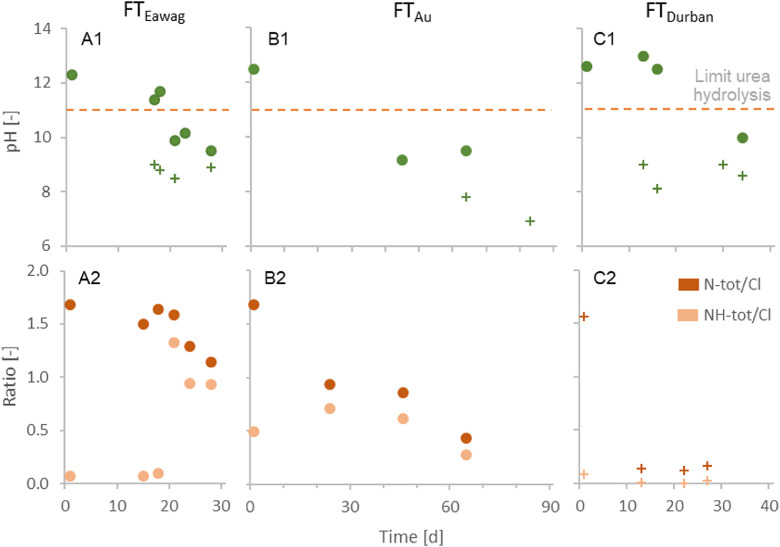
Development of pH (first row) and total nitrogen (N_tot_), respectively total ammonia (NH_tot_) to chloride (Cl) ratios (second row) inside the evaporation reactor during field tests FT_Eawag_, FT_Au_ and FT_Durban_. ● and + indicate measurements on the 1^st^, respectively the 6^th^ tray. The values at day 1 were measured in the overflow into the evaporation reactor (reference point).

In contrast to the results of the short-term evaporation tests, a substantial amount of nitrogen was lost during the long-term field tests (Fig. 4, see Table S.4 for data). After around 10 to 20 days, depending on the sampled tray, the pH dropped and urea hydrolysis started (Fig. 5). The recovery of nitrogen decreased to around 20% after one month (FT_Eawag_ and FT_Durban_) and even to 10% after three months (FT_Au_). The recovery efficiencies for the stabilization reactor, however, were close to 100% for FT_Eawag_ (92%) and FT_Au_ (94%) and 78% for FT_Durban_. The reason for the lower nitrogen recovery efficiency during stabilization in FT_Durban_ is unclear, since no substantial urea hydrolysis had occurred during stabilization (see Fig. 2). One reason could be that the values used for fresh urine in the calculation of FT_Durban_, were not representative: as it was not possible to measure the fresh urine during the field test, a once off collection campaign in the same household served as a basis.

**Fig. 6:**
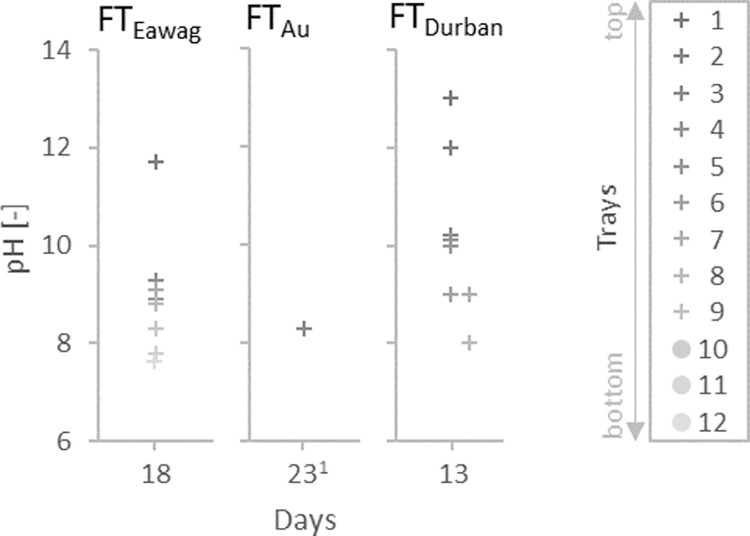
Exemplary pH gradients from top to bottom trays of the evaporation reactor during field tests FT_Eawag_, FT_Au_ and FT_Durban_ (only trays covered with urine appear in the graph). ^1^ Tray one could not be measured due to complete dry out.

The pH measurements on the trays (Fig. 5) showed a similar trend in all three field tests with values decreasing from initial pH values around 12.5 to values between 8 and 10 on all trays. Concomitant with the pH value, the nitrogen concentration given in relation to the tracer chloride decreased. In all experiments, the ratio of NH_tot_:Cl was much lower than the N_tot_:Cl ratio as long as the pH was above the critical value of 11 but close to the ratio of N_tot_:Cl for lower pH values. The strong increase of the NH_tot_ concentration can be explained by the biological urea hydrolysis, which can occur at pH values below 11 (Randall et al., 2016). In all field tests, the pH values were lower in the lower trays (Fig. 6), which means that the pH values decreased with the hydraulic retention time: the urine enters the reactor on the top and has to flow over all trays.

The lower pH values in the long-term experiments can be explained with the increased amounts of CO_2_ from the ambient air, which dissolved in urine and lead to a pH decrease. As a comparison, in the experiment Stab_Field_ 22 000 m^3^ of air was blown over the trays during the operation time, while in the field experiments, the air volumes ranged between 144 000 and 183 000 m^3^, being 6.5 to 8.3 times as high. A more detailed calculation of the CO_2_ absorption and the concomitant pH decrease would be beneficial, however, to do that it would be necessary to determine the exact kinetics of CO_2_ absorption in the urine module first.

According to Brison (2016), the strong pH decrease caused by CO_2_ dissolution is a combination of proton release from dissolved CO_2_, alkalinity consumption by CaCO_3_ precipitation, biological urea hydrolysis when the pH decreases and, finally, ammonia volatilization. Since urease and urease-producing bacteria were most probably deactivated during the stabilization phase (Geinzer 2017), it is likely that new urease was reintroduced to the evaporation system with ambient air during aeration.

The pH decrease due to CO_2_ dissolution can be delayed if residual Ca(OH)_2_ is available. However, this also means that CO_2_ dissolution consumes Ca(OH)_2_. Saving Ca(OH)_2_ was actually one of the reasons, why stabilization with Ca(OH)_2_ was separated from evaporation in this study. A single reactor system as operated by Dutta and Vinnerås (2016) and Senecal and Vinnerås (2017) has the advantage of a direct compensation of the introduced CO_2_ by Ca(OH)_2_ dissolution. However this comes at the cost of high consumption of Ca(OH)_2_ (see section 3.3.2).

Occasional malodor was reported for the field test FT_Au_. The cause were most likely volatile nitrogen or organic carbon compounds (Troccaz et al. 2013) and could be attributed to the hidden siphon discussed in section 3.1.1. While FT_Eawag_ and FT_Durban_ also had significant nitrogen loss, no malodor was reported. Online ammonia off-gas measurements in FT_Durban_ constantly showed values below the detection limit of 8 mg m^-3^ (see Figure S.8), which is below the Swiss maximum concentration at the workplace (long-term) of 14 mg m^-3^ (SUVA 2016). Following power cuts of more than 12 h, there were only two exceptions where ammonia concentrations at night time shortly reached 670 and 200 mg m^-3^ (10 min interval) inside the reactor. The low average ammonia concentration in the air can be validated based on the nitrogen mass balance. Dividing the nitrogen loss (250 g) by the total air throughput (183 000 m^3^) results in an average ammonia concentration in the air of 0.6 mg m^−3^. This calculation shows that there was a high dilution of the contaminant in the air and no risk for the users, even in case of a decreased stabilization rate. To reduce the emissions to the air as well as the nutrient loss, two measures can be taken: (i) increase the amount of residual Ca(OH)_2_ in the evaporation reactor, or (ii) reduce the CO_2_ introduced to the system. These measures are being considered in the further development of the technology.

#### 3.1.3 Phosphorus and potassium recovery

**Fig. 7:**
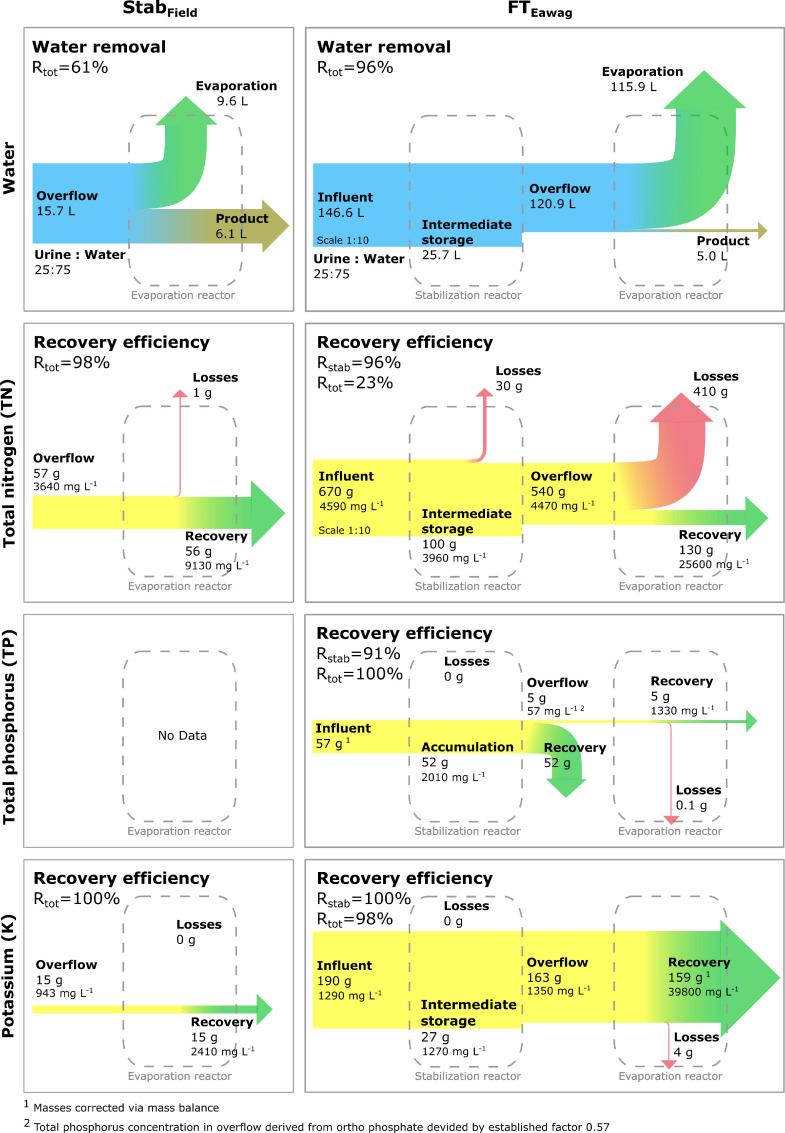
Overall mass flows of water and major nutrients being recovered in experiment Evap_Field_ and field test FT_Eawag_. FT_Eawag_ was run at 50% of its capacity. The part of the flow scheme not leaving the first system boundary is the fraction that is intermediately stored in the stabilization reactor.

Phosphorus and potassium were recovered close to 100% in the final products (Fig. 7). As there is no additional wastewater effluent leaving the system, the only loss occurred by incomplete removal of the products from the reactors. The design of the overflow between the stabilization reactor and the evaporation reactor led to a separation of nutrients resulting in two products: a calcium- and phosphate-rich precipitation product in the stabilization reactor and a viscous multi-nutrient product in the evaporation reactor. In the current setup, the precipitation product gets withdrawn at the bottom of the stabilization reactor and the multi-nutrient product is scraped of the trays after the remaining liquid fraction has been drained. In our experiments, 91% to 96% of the phosphate precipitated in the stabilization reactor, while the remaining part overflowed to the evaporation reactor. The separation of phosphorus is an advantage, as it can be blended to the mixed product according to the plant needs. As the recovery of nitrogen is a more complex topic, it is discussed separately in sections 3.1.1 and 3.1.2. During ongoing operation the drained urine from the trays will be reintroduced to the stabilization reactor, bringing the advantage that there is no waiting time until the urine is completely dried out and the urine module produces zero effluent for discharge, which could pollute the environment.

### 3.2 Water removal

During the long-term field test FT_Eawag_ about 96% of the incoming water was removed (Fig. 7). Overall, water removal was similar for the other long-term field experiments that is 92% for FT_Au_ and 93% for FT_Durban_. The removal rates, however, varied strongly. Between 0.54 and 1.28 kg_water_ m^−2^_wetted surface_ were removed in one day, when the fans were operated for 10 hours per day. The variations can be explained with differences in the climatic conditions (Table 4). Throughout all field tests the urine module never reached full capacity with the urine inflow being the limiting factor, which explains the low daily water removal. Maximum efficiency was thus just reached in the short term experiment Evap_Field_. Taken the evaporation rate determined in Evap_Field_ and extrapolating it to 12 trays and 24 h operation, the daily water removal would have been 19.8 kg d^−1^. This value is well above the design value of 13 kg d^−1^ for the Blue Diversion Autarky Toilet. However, it should be noted that at night, the RH is usually higher and the actual evaporation rate for 24 h operation would have been lower. Nevertheless, the capacity of the urine module is close if not above the design value. In areas prone to droughts it might be useful to include another step to recover the evaporated water. Water recovery, however, comes at the price of higher system complexity and higher energy demand.

**Table 4:** Mean evaporation rates and water removal at 10 h operation per day obtained in the long-term field tests FT_Eawag_, FT_Au_ and FT_Durban_ and in the short-term experiment Evap_Field_. Relative humidity (RH) and temperature are represented as the average values during the active operation hours (9:00-19:00) during the field tests.

	RH	Temperature	Evaporation rate	Available trays	Reactor capacity used	Daily water removal (10h operation)
	[%]	[°C]	[g m^−2^ h^−1^]		[# trays]	[kg d^−1^]
Evap_Field_	54±24	20±8	131±44	4	4 (100%)	2.76±1.31
FT_Eawag_	60±19	24±5	128^1^	12	6.0 (51%)	4.16
FT_Au_	82±13	12±6	54^1^^,^^2^	12	1.3 (8%)	0.28
FT_Durban_	75±12	21±4	79^1^^,^^2^	12	6.8 (60%)	2.98

1 Values calculated via total input-output balance during one harvest period.

2 Input values calculated with tracer load (chloride, Equation 3).

**Fig. 8:**
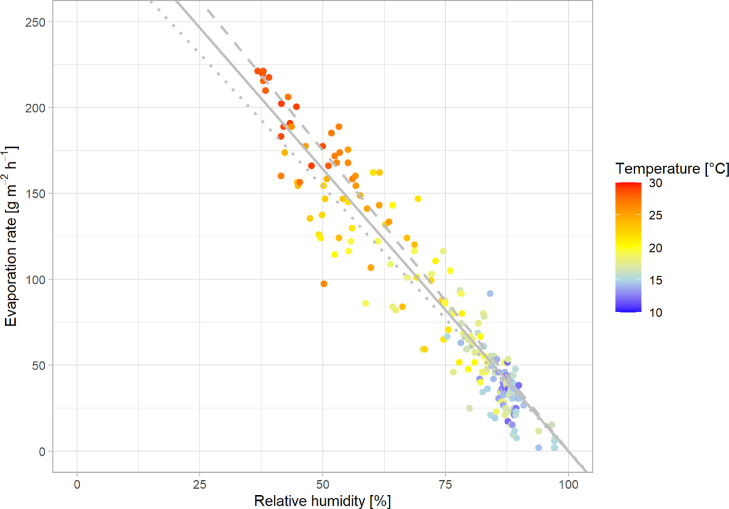
Evaporation rate as a function of relative humidity (RH) and temperature in the incoming air measured in experiment Evap_Field_. Higher temperature and lower RH result in a higher evaporation rate. The regression lines were fitted with the evaporation and RH data at the temperatures 10°C (dotted), 20°C (line) and 30°C (dashed).

The experiment Evap_Field_ showed that the evaporation rate of water from stabilized urine correlated with the ambient RH (Fig. 8). Furthermore, the evaporation rate depended on the ambient temperature. At higher temperatures, the water holding capacity of the air is increased. Consequently, higher temperatures at the same RH lead to an increased evaporation rate. Also the mass transfer from the water to the gas phase increases with the temperature, in accordance with the increase of the diffusion coefficient of water vapor in air. The measured data could be described sufficiently well with a model according to Equation 11, which considers the difference between 100% and the measured RH in the ambient as driving force and a mass transfer coefficient which depends on the temperature in the incoming air (Fig. 8)

(11) $$E=k_{m,T_{0}}\cdot e^{0.0063\cdot \left(T-T_{0}\right)}\cdot \left(1-\mathrm{RH}\right)\left[g\cdot m^{-2}\cdot h^{-1}\right]$$

With

E: evaporation rate

$k_{m,T}=k_{m,T_{0}}\cdot e^{b\cdot (T-T_{0})}$: mass transfer coefficient [g m^−2^ h^−1^]

$k_{m,T_{0}}$=3.29 g m^−2^ h^−1^: mass transfer coefficient at T_0_

$e^{b\cdot (T-T_{0})}$: temperature correction assuming diffusion depends exponentially on temperature [-]

b=0.0063: temperature correction exponent [°C^−1^] interpolated from values for water vapor diffusion in air (Lide 2009)

T: temperature [°C]

T_0_=20°C: reference temperature

1−RH: driving force for evaporation [-]

RH:relativehumidity [-]

 

The basic assumptions of Equation 11, however, are not correct according to the theory of evaporation. The driving force is actually the difference between the actual and the maximum achievable absolute humidity of the air in the reactor. Furthermore, the relevant temperature is the temperature at the water/air interface (Bergman et al. 2011). Since these data were not available to us, we used relative humidity and temperature measurements of the incoming air. Apparently, using the temperature and RH of the incoming air compensate for the deviations from theory. A fit with the absolute humidity of the incoming air did result in worse correlations (see residuals in Figure S.9 and Figure S.10). The approach used in Equation 11 might therefore be useful in practice, when absolute humidity in the reactor and the temperature at the water/air interface cannot be easily determined as it was the case in our experiments.

The average evaporation rates for the urine module (Table 4) compare favorably with results from previous studies: Based on the linear fit of the experimental data (Evap_Field_) at 70% RH and 20°C, the urine module would evaporate around 94 g m^−2^ h^−1^. It shows that a system operated at a high airflow can compensate for the high RH. For comparison, Bethune et al. (2016) reported 110 g m^−2^ h^−1^ at 22% RH and 20°C. At the same conditions of 70% RH and 20°C, Dutta and Vinnerås (2016) evaporated 8 g m^−2^ h^−1^. Both research groups operated at low airflows per evaporation surface, 5.5 m^3^ m^−2^ h^−1^ and 1.7 m^3^ m^−2^ h^−1^, respectively, which were substantially lower than the 130 m^3^ m^−2^ h^−1^ in our experiments. Another possibility to increase evaporation is heating the incoming air. By using simple solar dehydration, Antonini et al. (2012) achieved 42 g m^−2^ h^−1^ (RH not provided, 50-60°C). During short-term tests with pre-heating the air to 50°C and with 20% RH Simha et al. (2018) achieved a very high evaporation rate of 500 g m^−2^ h^−1^. However, heating results in substantially higher energy demands of 5 to 30 times compared to the use of high airflow at ambient temperature (see discussion in section 3.3.1).

### 3.3 Resource requirements

#### 3.3.1 Energy

The average daily electrical energy demand for the urine module tested in the field (FT_Durban_) was 1260±270 Wh d^−1^ and corresponded well with the expected demand of 1354 Wh d^−1^ (stirrer in the stabilization reactor: 58 Wh d^−1^ and fans in the evaporation reactor: 1296 kWh d^−1^). As the two energy consuming appliances are operated independently of the urine inflow, the relative energy demand per kg water evaporated was determined by the urine inflow, i.e. the used reactor capacity, and on the evaporation rates, which in turn depended on the RH of the incoming air (see Fig. 8). A comparison of different reactor settings and their specific energy demands is given in Table 5.

**Table 5:** Specific energy demand for different urine dehydration systems

	BDAT urine module^1^				Alkaline dehydration in ash		Distillation
Setting	Evap_Field_^1^	FT_Eawag_^1^	FT_Au_^1^	FT_Durban_^1^	Laboratory	Field	Field
Trays inside reactor	4	12	12	12	-	-	-
Reactor capacity used^2^ [%]	100	50	10	56	100	∼20	100
Spec. energy demand [Wh kg_H2Oevap_^−1^]	150	300	4520	420	1200–3200	24500	110
Cost^3^ [EUR pers^−1^ yr^−1^]	18	36	537	50	143-380	2910	13
Reference	This study				(Senecal 2020)	(Simha et al. 2020a)	(Fumasoli et al. 2016)

1 Same basic reactor setting for all tests, including use of activated carbon air filter mat.

2 Percentage of total tray area covered with urine.

3 Calculated with EU electricity mix (0.217 EUR kWh^−1^), assuming 1.5 L urine pers^−1^ d^−1^ and all water removed.

The specific energy demand of our system is much lower than the theoretical value of 710 Wh kg_H2Oevap_^−1^ required for water evaporation by heating (Udert and Wächter 2012). In the experiment Evap_Field_ it was actually close to 110±30 Wh kg_H2Oevap_^−1^, which was reported for water removal from nitrified urine in a distiller with vapour compression and heat recovery (Fumasoli et al. 2016). While in the system used by Fumasoli et al. (2016), the low energy demand was achieved by recovering heat during vapour condensation, the lower energy demand of our system can be explained by capturing heat from the ambient air, which is cooled during the evaporation process. This is supported by measurements during FT_Au_, showing an average temperature difference of 0.7±1.1°C between air in- and outflow (at an average T_in_ = 12±6°C). A comparison in Table 5 suggests that air pre-heating (65°C, Senecal 2020) without heat recovery at a low air flow (∼30 m^3^ h^-1^ m^-2^, Simha et al. 2018) may be energetically less favourable, by at least a factor of 10, than increasing the air flow (130 m^3^ h^−1^ m^−2^, this study) using ambient air. A disadvantage of a high air flow is the faster dissolution of CO_2_ from the air, leading to a pH decrease and subsequently to enzymatic urea hydrolysis as discussed in section 3.1.2. The use of heat, however, favours chemical urea hydrolysis.

For an efficient energy use, it is important to know the amount of urine inflow and thus the used reactor capacity. Due to its modular structure the urine module can be adjusted to three different programs, using 3 fans with 4 trays, 6 fans with 8 trays or 9 fans with 12 trays. A sophisticated process control, taking into account the urine inflow, could also strongly reduce the energy demand. However, such a process control needs to be sufficiently robust for the challenges of on-site applications in locations with low technical service. Further energy reduction potential lies in the reduction of friction for the fans. The activated carbon air filter mat did not show a high benefit during the field tests, as it could not prevent a malodour event in FT_Au_. By removing it, the consumed energy could be reduced by 17% (from 420 to 350 Wh kg_H2Oevap_^−1^) during FT_Durban_. Removing the filter also increased the airflow from 800 to 1050 m^3^ h^−1^ (Figure S.11), which is close to the originally aimed design value (1100 m^3^ h^−1^) and increased the evaporation efficiency.

#### 3.3.2 Additives

Our experiments showed that 6 g_Ca(OH)2_·L_urine_^−1^ are sufficient for urine stabilization. This is lower than the 10 g·L^−1^ suggested by Randall et al. (2016), who, however, considered a safety factor of 2. When using 6 g·L^−1^ around 22 kg of Ca(OH)_2_ per year would be necessary for a treatment rate of 10 L_urine_ d^−1^. Compared to other reported on-site urine treatment systems, for which acid or base was used for urine stabilization, this is a rather small amount of additive and the costs are low (Table 6).

**Table 6:** Comparison of required additive amounts and costs of different small-scale urine treatment systems

	Ca(OH)_2_ treatment	Mixed ash & lime treatment	Alkaline ash treatment	Magnesium treatment	Acid treatment 1	Acid treatment 2
Additive	Ca(OH)_2_	Wood ash, Ca(OH)_2_ (1:1)	Wood ash	Mg(OH)_2_, MgCl_2_ (3:1)	Sulphuric acid (96%)	Phosphoric acid (89%)
Dosing	Passive^1^	Passive^1^	Passive^1^	Passive^1^	Active^2^	Active^2^
Spec. amount [g·L_urine_^−1^]	6	100-290^3^	50^3^	22	27.76 [mL·L^−1^]	31.46 [mL·L^−1^]
Annual amount^4^ [kg yr^−1^]	22	365-1058	183	80	101 [L yr^−1^]	115 [L yr^−1^]
Cost^5^ [EUR pers^−1^ yr^−1^]	0.20-0.70	2-17^6^	0^6^	1-25	61-390	9-108
Reference	This study	(Dutta and Vinnerås 2016)	(Senecal and Vinnerås 2017)	(Vasiljev et al. 2022)	(Antonini et al. 2012)	(Antonini et al. 2012)

1 Reservoir of solids with low solubility inside reactor.

2 External liquid dosing device.

3 Calculated based on total amounts of additive per volume urine applied.

4 Calculated assuming urine treatment of 10 L d^−1^ during 365 d.

5 All costs displayed show an order of magnitude; the price calculation (Table S.5) varies significantly according to origin and purchased entity.

6 Assuming ash is a waste product and thus adds no costs.

Adding up the operational costs of 18 EUR pers^−1^ yr^−1^ for electricity and 0.70 EUR pers^−1^ yr^−1^ for Ca(OH)_2_ we end up at daily costs of 0.05 EUR pers^−1^ d^−1^. This value is in the same range as the 0.05 USD pers^−1^ d^−1^, which was set as challenging task by the Bill and Melinda Gates Foundation for the overall costs of non-sewered sanitation systems. The overall costs, however, would be higher for the urine module and even more for the whole BDAT. A major cost factor not considered for the urine module could be the service by a technician. However, selling fertilizer might result in some revenue. This revenue cannot be quantified at the moment, because the costs for a possible post-treatment of the fertilizer and the market price of the final product are not known yet.

### 3.4 Fertilizer quality

#### 3.4.1 Nutrient availability for plants

In a previous study by Meyer et al. (2017), the plant availability of phosphorus in alkaline soils was tested for two possible products from our process: the precipitation product from the stabilization reactor and a blend of the two products from the stabilization and the evaporation reactor. Both potential fertilizers were produced from real urine in the laboratory. The plant availability of phosphorus was 22 and 22.9 mg_P_ kg_Soil_^−1^, for the stabilization reactor product and the blended product, respectively. These values were very similar to the values for water soluble phosphorus (23.9 mg_P_ kg_Soil_^−1^) and around 30% higher than for sewage sludge ash (15.3 mg_P_ kg_Soil_^−1^). These results were promising, but further studies on the plant availability of nutrients besides phosphorus and on possible effects of salt and unwanted substances such as pharmaceuticals are needed for an overall assessment of the fertilizer quality of the products from the urine module. Depending on the results of this assessment, further processing might be necessary.

#### 3.4.2 Pathogen removal

Pathogens testing at the end of one harvest cycle in FT_Durban_ suggests that there is no contamination with fecal bacteria in the concentrated evaporation product: E. coli and total coliforms were both below 1 MPN (most probable number)/100 mL. Furthermore, no helminths were detected in the evaporation product, and only one undeveloped Ascaris suum egg was determined in a 500 mL sample of the stabilization reactor (see Table S.6). To be certain that helminths are effectively removed, spiking tests are suggested, as the naturally applied helminth load during the field tests is unknown. While for bacteria and viruses, Senecal et al. (2018) confirmed a complete inactivation after four days in their study on urine stabilized with base at a pH of above 10.5 (20°C), it took 166 days to reach 3 log_10_ reductions of helminths (*Ascaris suum*). At a temperature of 42°C, the time to reach 3 log_10_ reductions was reduced to nine days. As we run our system at pH 12.5, it is advised to redo spiking tests for this pH level. However, the majority of helminths are excreted with feces. To comply with hygiene standards of the WHO (2006) and USEPA (1994) for human waste treatment, source separation alone reaches a 5.2 log_10_ reduction of Ascaris suum (Senecal et al. 2018).

## 4 Conclusions

• Short-term evaporation experiments lasting four days showed that urea hydrolysis can be prevented successfully by stabilizing urine at a pH around 12.5 with Ca(OH)_2_. However, in long-term field tests, a pH decrease to values below 11 resulted in a significant nitrogen loss. CO_2_ absorption is most probably driving the pH decrease, which in turn results in biological urea hydrolysis. To tackle this issue, two measures can be taken: (a) allow excess Ca(OH)_2_ to enter the evaporation reactor, or (b) regulate the CO_2_ input through the air.
• Water could be removed reliably at ambient air temperature without additional heating by just using a high airflow as the main driver. The water removing capacity is closely linked to the RH of the incoming air. At 20°C, 55% RH and 24h operation of the fans, the water removing capacity would be 20 kg d^−1^, which is substantially above the target value of 13 kg d^−1^. If operated outside, low temperatures and high RH at night can reduce the water removing capacity.
• The operational costs are expected to be reasonably low. The energy and the additive demand resulted in estimated costs of 0.05 EUR pers^−1^ d^−1^. The performance of the system and thereby the operational cost strongly depended on (a) the used reactor capacity and (b) the RH of the incoming air.
• Phosphate was recovered as a separate product from the stabilization reactor. Depending on the market demand, this product could be used independently or be blended with the second mixed nutrient product from the evaporation reactor or other fertilizers. Revenues from fertilizers could offset operational costs.

## CRediT authorship contribution statement

**Michel E. Riechmann:** Conceptualization, Methodology, Investigation, Formal analysis, Writing – original draft, Writing – review & editing, Visualization. **Bonginkosi Ndwandwe:** Investigation, Writing – review & editing. **Esther E. Greenwood:** Investigation, Writing – review & editing. **Eva Reynaert:** Investigation, Writing – review & editing. **Eberhard Morgenroth:** Writing – review & editing, Supervision. **Kai M. Udert:** Conceptualization, Writing – review & editing, Supervision, Project administration, Funding acquisition.

## Declaration of Competing Interest

The authors declare that they have no known competing financial interests or personal relationships that could have appeared to influence the work reported in this paper.

## Appendix B Supplementary materials

Image, application 1
