# Supplementary material for: On-site urine treatment combining Ca(OH)2 dissolution and dehydration with ambient air
Source: Water Res X. 2021 Oct 8;13:100124. doi: 10.1016/j.wroa.2021.100124 (PMC8645517; doi:10.1016/j.wroa.2021.100124)
Supplement: Supplementary file 1 [file mmc1.pdf]

Supplementary Information for

On-site urine treatment combining $Ca(OH)_2$ dissolution and dehydration with ambient air

Michel E. Riechmann [a], Bonginkosi Ndwandwe [c], Esther E. Greenwood [a],

Eva Reynaert [a, b], Eberhard Morgenroth [a, b], Kai M. Udert [a, b,*]

[a] *Eawag, Swiss Federal Institute of Aquatic Science and Technology, 8600 Dübendorf, Switzerland*

[b] *ETH Zürich, Institute of Environmental Engineering, 8093 Zürich, Switzerland*

[c] *University of KwaZulu Natal, WASH R&D Center, 4041 Durban, South Africa*

Water Research X (2021), Article No 100124, https://doi.org/10.1016/j.wroa.2021.100124

* *Corresponding author:* *Kai M. Udert, Kai.Udert@eawag.ch*
Eawag, Swiss Federal Institute of Aquatic Science and Technology,
Department of Process Engineering,
8600 Dübendorf, Switzerland

Contents

S.1 Photo documentation of field testing settings 3

S.2 Detailed setting of field tests 4

S.3 Climate conditions 5

S.4 Sample analysis 6

S.5 Results experiments $Stab_{Lab}$ and $Stab_{Field}$ 8

S.6 Nitrogen recovery calculation data 9

S.7 Ammonia off-gas measurement 11

S.8 Water removal 11

S.9 Air flow 13

S.10 Additive cost calculations 14

S.11 Pathogen testing 15

S.1 Photo documentation of field testing settings

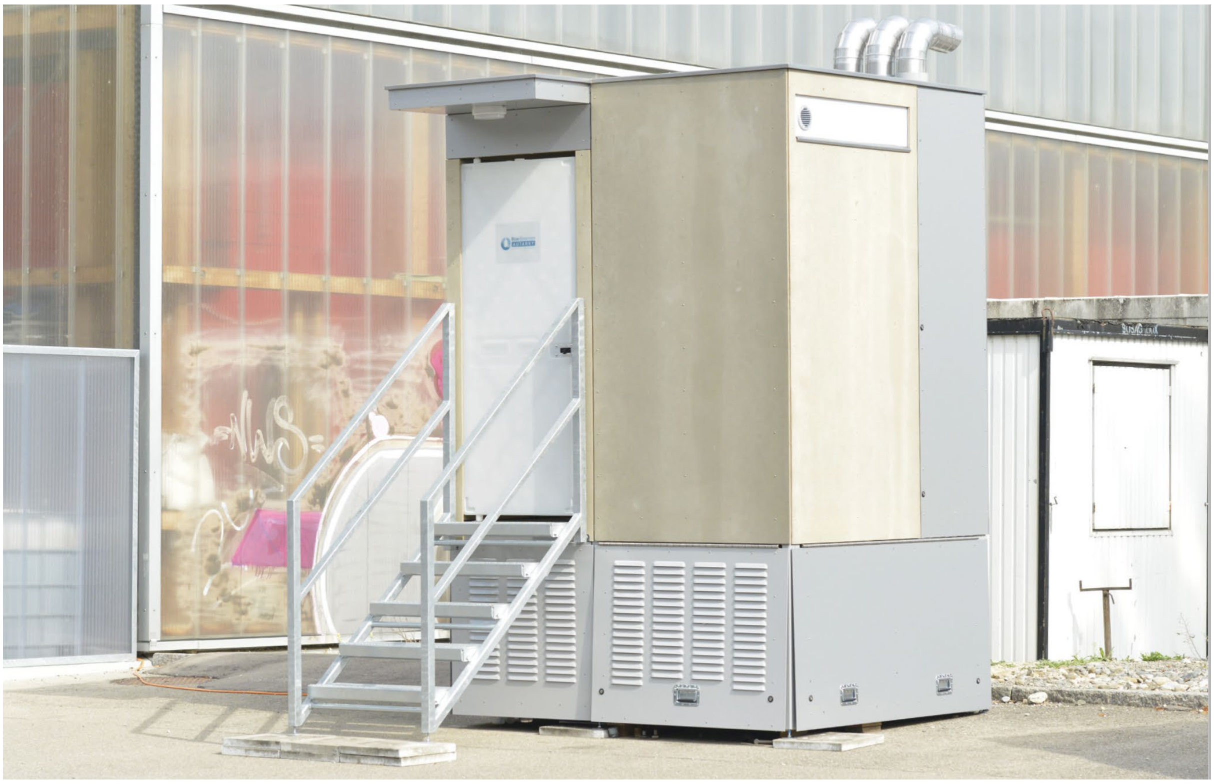

Figure S.1: Setting of the field test and complementing experiments with the urine module as part of the Blue Diversion Autarky Toilet next to Eawag experimental hall

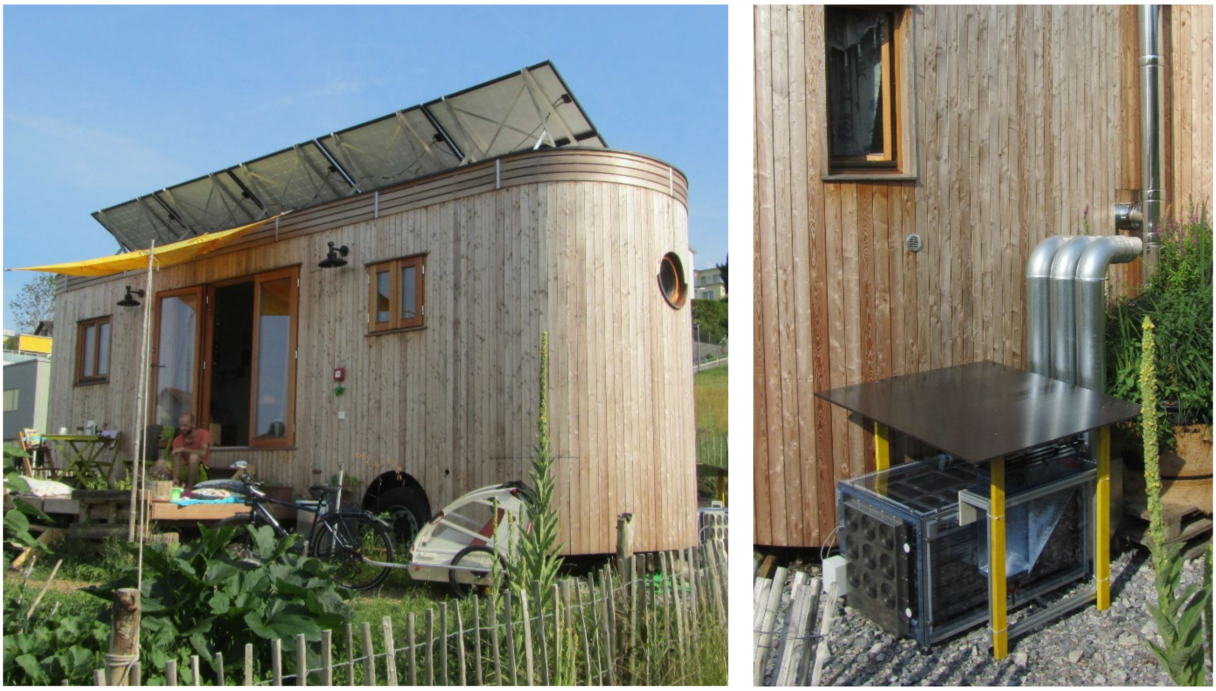

Figure S.2: The Autarky urine module attached to a tini house in Au (Zurich), Switzerland

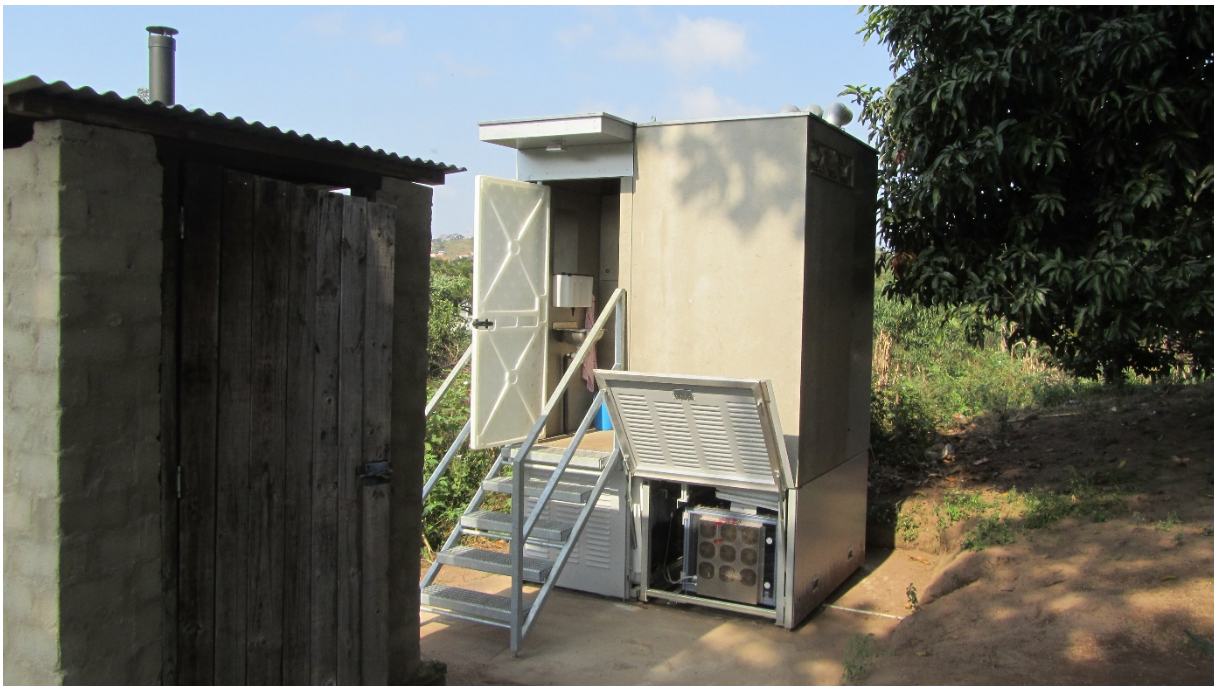

Figure S.3: The Blue Diversion Autarky Toilet with the urine module below the cabin during field tests in a peri-urban settlement in Durban, South Africa

S.2 Detailed setting of field tests

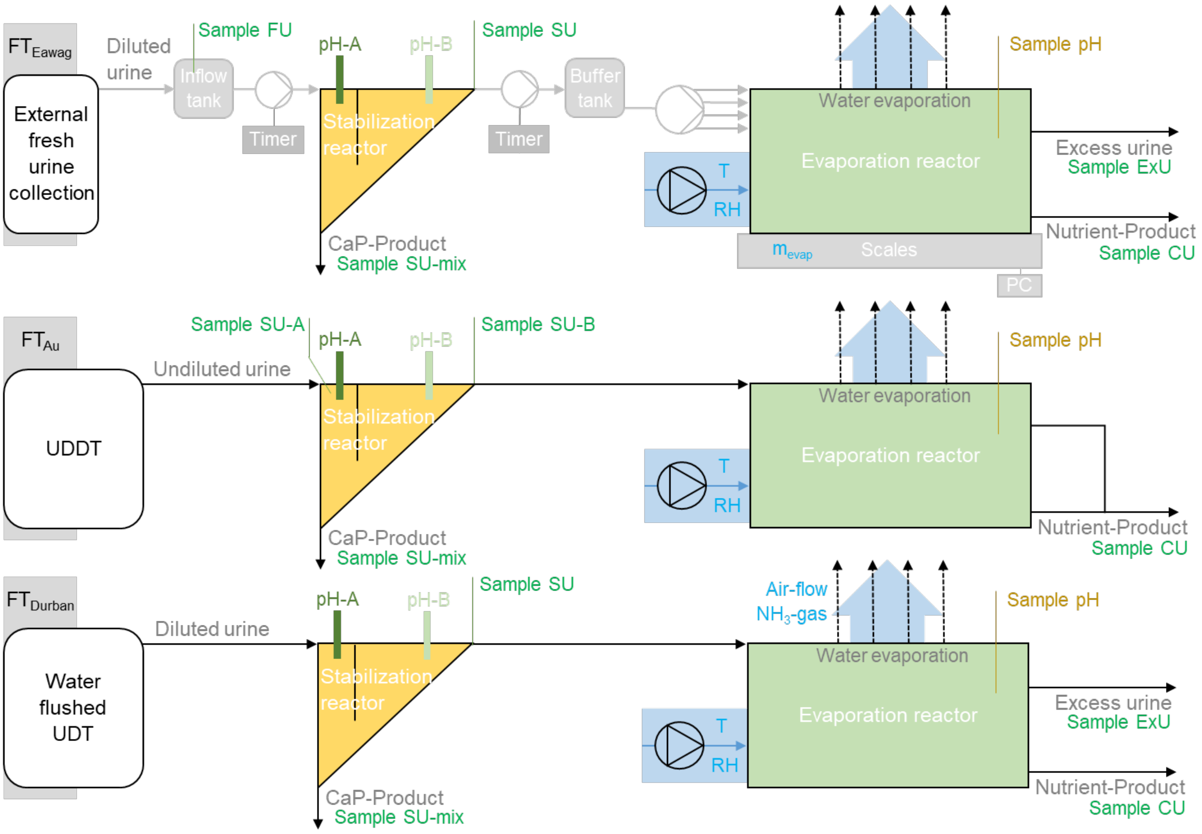

Figure S.4: Experimental set-up of field test FT_{Eawag}, FT_{Au} and FT_{Durban}.
UD(D)T: Urine diversion (dry) toilet; FU: Fresh urine; SU: calcium hydroxide stabilized urine; ExU: Excess urine; CU: Concentrated urine; pH-A/B: Online pH log; T: Temperature; RH: Relative humidity; NH_3-gas: Ammonia measurement in off-gas; m_{Evap}: Online mass log

S.3 Climate conditions

Table S.1: Climate conditions during the field tests FT_{Eawag}, FT_{Au} and FT_{Durban} compared to the planned working ranges of the urine treatment module

			Working range	FT_{Eawag} 9-21h	FT_{Au} 9-21h	FT_{Durban} 8-20h
Relative humidity [%]	Average	RH_{min}		24	29	21
		RH_{max}		95	98	96
		$RH_{average}$		70	84	84
	Day	RH_{min}	20	28	39	21
		RH_{max}	80	94	98	96
		$RH_{average}$	-	60	81	77
	Night	RH_{min}		45	29	53
		RH_{max}		95	98	96
		$RH_{average}$		80	88	91
Temperature [°C]	Average	T_{min}	0	12	0	5
		T_{max}	40	36	31	34
		$T_{average}$	-	21	11	18
	Day	T_{min}		15	0	7
		T_{max}		36	31	3491
		$T_{average}$		24	12	21
	Night	T_{min}		12	1	5
		T_{max}		30	28	24
		$T_{average}$		18	10	16

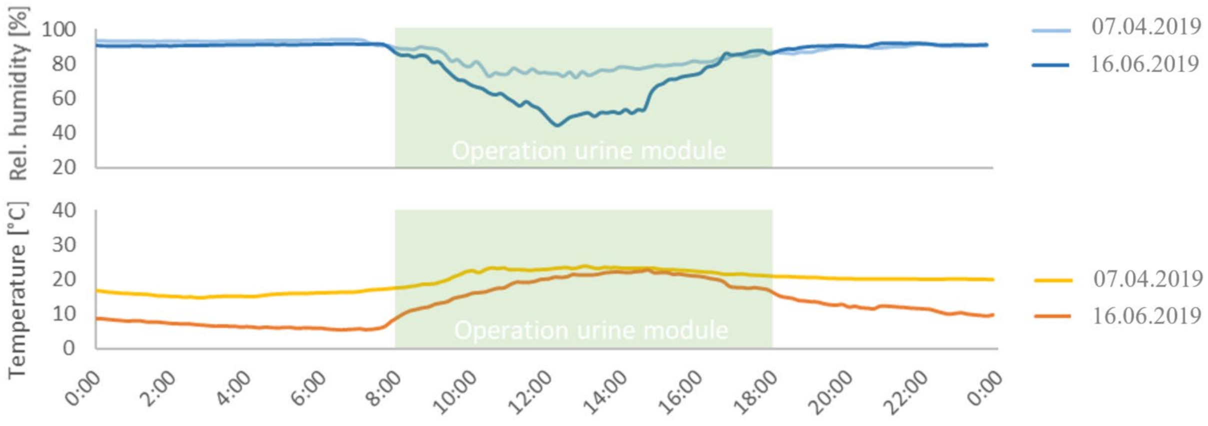

Figure S.5: Two typical daily profiles from the field tests in FTDurban show the variance of relative humidity (RH) and temperature throughout two exemplary days. The first profile shows a constant, similarly high RH during night time and a differing of the two days during the day. The second graph instead shows an approximation of the T during the day and a divergence at night. Both, the T and the RH gradient of the 07.04.2019 are lower than those of 16.06.2019.

S.4 Sample analysis

Table S.2: Test methods and sampling frequencies during FT_{Eawag}, FT_{Au} and FT_{Durban}

Parameter	**Method** $\mathbf{FT_{Eawag}}$ **&** $\mathbf{FT_{Au}}$	**Interval** $\mathbf{FT_{Eawag}}$ **(**$\mathbf{FT_{Au}}$**) [#/week]**	**Method** $\mathbf{FT_{Durban}}$	**Interval** $\mathbf{FT_{Durban}}$ **[#/week]**
Total chemical oxygen demand (COD_{tot})	-	-	APHA 5220D-COD Closed Reflux, Colorimetric Method, Photometer DR900 (Dichromate), Hach, Düsseldorf, Germany	1x-2x
Total organic carbon (TOC)	TOC-L, Shimadzu, Kyoto, Japan	2x (1x)	-	-
Total nitrogen (N_{tot})	TOC-L, Shimadzu, Kyoto, Japan	2x (1x)	Spectroquant NOVA 60 (Peroxodisulfate oxidation/2,6-Dimethylphenol), Merck, Darmstadt, Germany	3x, later 2x
Urea-N	Lachat QC8500 FIA, Hach, Düsseldorf, Germany; (via SOP and NH_{tot} detection)	2x (1x)	-	-
Nitrate (NO_3-N)	-	-	Spectroquant Prove 300 (2,6-Dimethylphenol), Merck, Darmstadt, Germany	1x
Nitrite (NO_2-N)	-	-	Spectroquant Prove 300 (Griess reaction), Merck, Darmstadt, Germany	1x
Total Ammonia (NH_{tot}-N)	Lachat QC8500 FIA, Hach, Düsseldorf, Germany	2x (1x)	Spectroquant Prove 300 (Indophenol blue), Merck, Darmstadt, Germany	3x, later 2x
Total Phosphorus (P_{tot})	-	-	Spectroquant Prove 300 (Peroxidsulfate oxidation phosphormolybdenum blue), Merck, Darmstadt, Germany	3x, later 1x
Ortho-phosphate (PO_4-P)	930 Compact IC (Ion Chromatography) Flex, Metrohm, Herisau, Switzerland	2x (1x)	-	
Sulphur (SO_4)	930 Compact IC Flex, Metrohm, Herisau, Switzerland	2x (1x)	-	

Chloride (Cl)	930 Compact IC Flex, Metrohm, Herisau, Switzerland	2x (1x)	Spectroquant Prove 300 (Iron(III)-thiocyanat), Merck, Darmstadt, Germany	3x, later 2x
Sodium (Na)	930 Compact IC Flex, Metrohm, Herisau, Switzerland	2x (1x)	Spectroquant Prove 300 (Iron(III)-thiocyanat), Merck, Darmstadt, Germany	3x, later 2x
Potassium (K)	930 Compact IC Flex, Metrohm, Herisau, Switzerland	2x (1x)	Spectroquant Prove 300 (Kalignost®, turbidimetric), Merck, Darmstadt, Germany	3x, later 2x
Calcium (Ca)	930 Compact IC Flex, Metrohm, Herisau, Switzerland	2x (1x)	Spectroquant Nova 60, Merck, Darmstadt, Germany	1x
Magnesium (Mg)	930 Compact IC Flex, Metrohm, Herisau, Switzerland	2x (1x)	Spectroquant Nova 60 (phthalein purple), Merck, Darmstadt, Germany	occasionally

Helminths testing procedure

(Laboratory Wash R&D Center, formerly Pollution Research Group, University of KwaZulu-Natal, Durban, South Africa)

Each sample was well mixed and filtered through a 100µm mesh sieve that was placed on top of a 20µm mesh sieve. The sample bottle was rinsed three times with tap water that was also poured through the two sieves. The filters were then washed with tap water using a hose attachment. The retentate caught on the 100µm sieve was discarded and the retentate on the 20µm sieve was collected and transferred into one or two 15ml plastic conical test tubes that were then centrifuged at 3000rpm (1389g) for 10 minutes. The supernatant was discarded, and the entire pellet was pipetted onto one or more microscope slides, a coverslip was placed on top, and the preparation was examined under a compound microscope with 10x and 40x objectives. Any helminth eggs seen were counted and assessed as potentially viable or dead.

S.5 Results experiments $Stab_{Lab}$ and $Stab_{Field}$

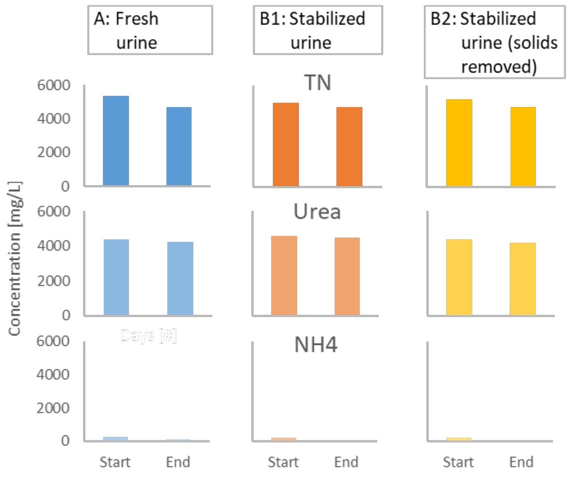

Figure S.6: Fate of total nitrogen (TN), urea and ammonium (NH_4) in fresh, stabilized and stabilized with solids removed urine during the laboratory experiment $Stab_{Lab}$

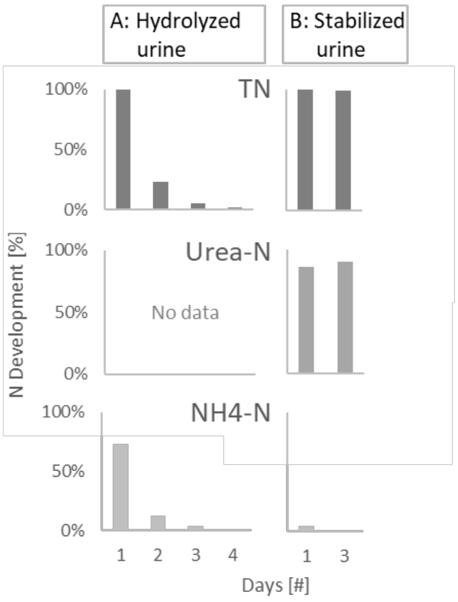

Figure S.7: Comparison of nitrogen stability in hydrolyzed (starting pH: 9) and calcium hydroxide stabilized urine (starting pH: 12.5) during active ventilation in the urine module (experiment $Stab_{Field}$). The values starting from day two were normalized (with tracer Cl) to account for concentration distortion induced by the water evaporation.

S.6 Nitrogen recovery calculation data

Table S.3: Measured ions and calculations for nitrogen (N) development during experiment $Stab_{Field}$

	Date	Day	Ions			
			**TN [mg N/L]**	**NH4 [mg N/L]**	**Urea [mg N/L]**	**Cl [mg/L]**
A: Hydrolysed urine (Evaporation reactor, tray 2)	11.02.2019	1	6300	4600	n.d.	3100
	12.02.2019	2	2000	1100	n.d.	4200
	13.02.2019	3	400	300	n.d.	4000
	14.02.2019	4	200	140	n.d.	5300
B: Ca(OH)2 stabilized urine (Evaporation reactor)	13.09.2019	1	3636	137	3114	2583
	16.09.2019	3	11849	31	8269	13910
	Correction factor (tracer Cl)					2.5
	Corrected outflow values (tracer Cl)		3599	12	3260	

	Date	Day	Tracer ratios			Percent Recovery (Cl-corrected)		
			**TN/Cl**	**NH4/Cl**	**Urea/Cl**	**TN [%]**	**NH4 [%]**	**Urea [%]**
A: Hydrolysed urine (Evaporation reactor, tray 2)	11.02.2019	1	2.03	1.48	n.d.	100%	73%	n.d.
	12.02.2019	2	0.48	0.26	n.d.	23%	13%	n.d.
	13.02.2019	3	0.10	0.08	n.d.	5%	4%	n.d.
	14.02.2019	4	0.04	0.03	n.d.	2%	1%	n.d.
B: Ca(OH)2 stabilized urine (Evaporation reactor)	13.09.2019	1	1.41	0.05	1.21	100%	4%	86%
	16.09.2019	3	1.39	0.00	1.26			
	Correction factor (tracer Cl)							
	Corrected outflow values (tracer Cl)					99%	0%	90%

Table S.4: Data used for N recovery calculation in Figure 4 (Paper)

	**Ntot**	**Cl**	**Ntot/Cl**	**Recovery**		**Fractions**
FT_{Eawag}	**[mgN L-1]**	**[mg L-1]**	**[-]**	**[%]**		**[%]**
Inflow	4225	2239	1.89		Loss stab. reactor	8%
SD	1175	273	0.43			
Stabilization reactor	4468	2590	1.73	92%	Loss evap. reactor	72%
SD	631	284	0.17	23%		
Evaporation reactor	25640	69943	0.37	19%	Final recovery	19%
SD	671	1280	0.01	4%		
FT_{Au}						
Inflow	5884	3285	1.79		Loss stab. reactor	6%
SD	505	125	0.09			
Stabilization reactor	6002	3573	1.68	94%	Loss evap. reactor	87%
SD	471	223	0.06	6%		
Evaporation reactor	12955	100423	0.13	7%	Final recovery	7%
SD	276	2117	0.00	0%		
FT_{Durban}						
Inflow	9256	4690	1.97		Loss stab. reactor	22%
SD	82	73	0.04			
Stabilization reactor	2082	1358	1.53	78%	Loss evap. reactor	62%
SD	180	124	0.11	6%		
Evaporation reactor	5918	19285	0.31	16%	Final recovery	16%
SD	491	1961	0.04	2%		

S.7 Ammonia off-gas measurement

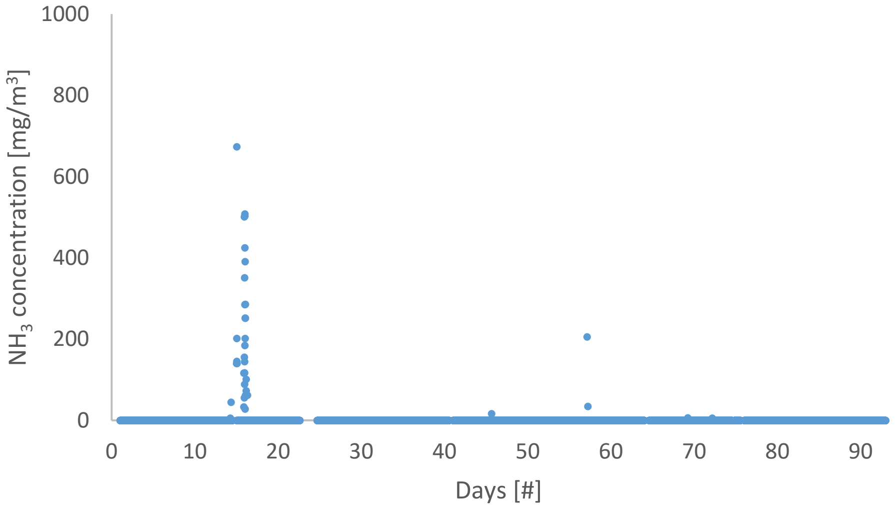

Figure S.8: Ammonia (NH_3) measurement in the off-gas of the urine module during FT_{Durban}

S.8 Water removal

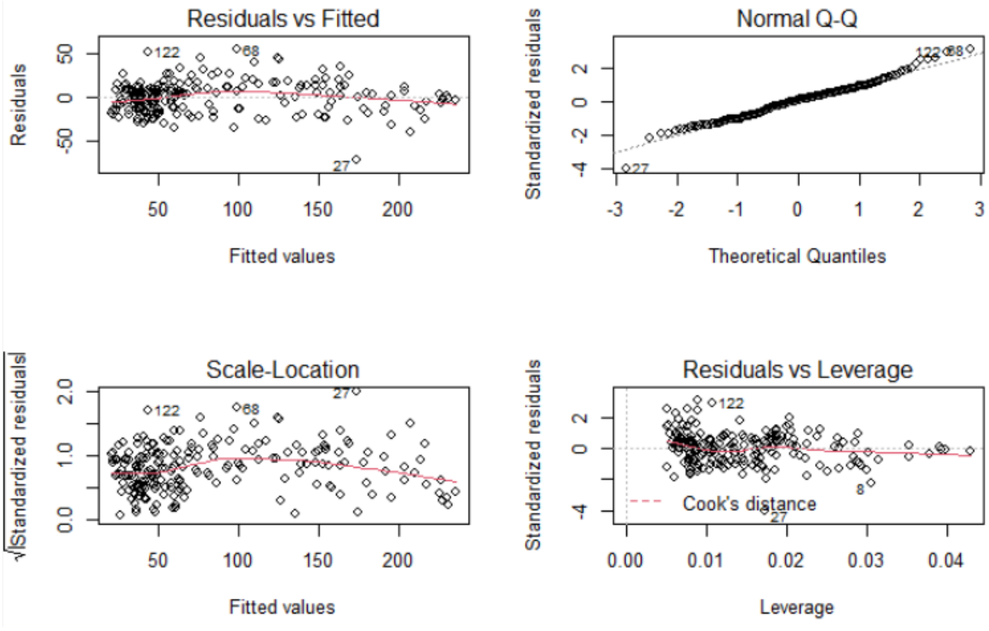

Figure S.9: Residuals produced by linear regression using the evaporation model (Equation 11 in paper with RH_{max}-RH as a driving force fitted) with data from experiment $Stab_{Field}$

```
Residuals:
    Min      1Q  Median      3Q     Max 
-68.010 -11.469   0.401   9.456  52.508 

Coefficients:
                     Estimate Std. Error t value Pr(>|t|)    
EvaporationfactorRH  3.28555    0.03924   83.72   <2e-16 ***
---
Signif. codes:  0 ‘***’ 0.001 ‘**’ 0.01 ‘*’ 0.05 ‘.’ 0.1 ‘ ’ 1

Residual standard error: 17.64 on 209 degrees of freedom
Multiple R-squared:  0.971,	Adjusted R-squared:  0.9709 
F-statistic:  7010 on 1 and 209 DF,  p-value: < 2.2e-16
```

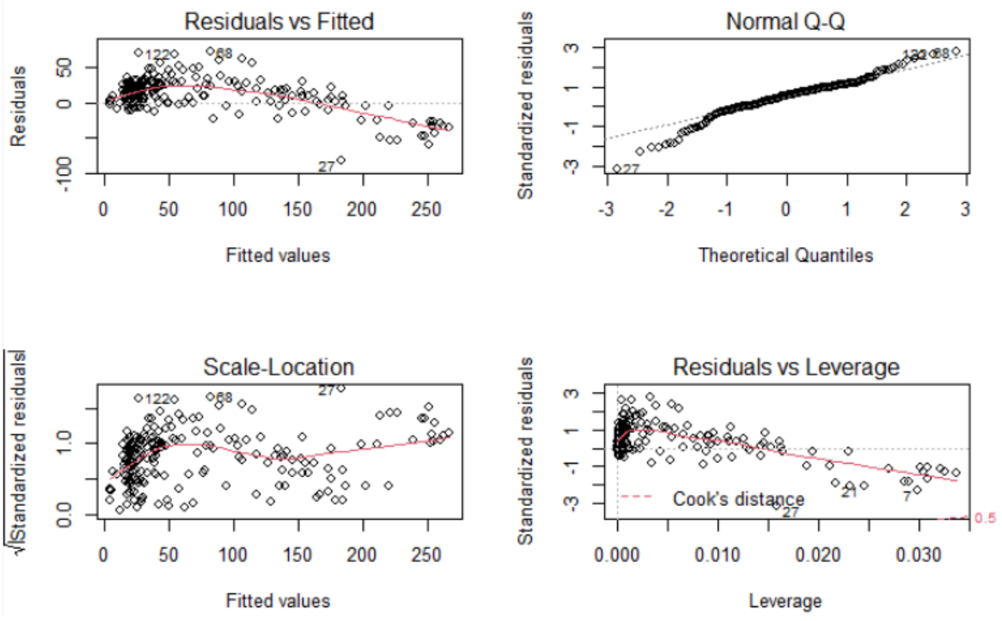

Figure S.10: Residuals produced by linear regression using the evaporation model (Equation 11 in paper with AH_{sat}-AH as a driving force fitted) with data from experiment $Stab_{Field}$

```
Residuals:
    Min      1Q  Median      3Q     Max 
-80.800   0.801  14.678  25.352  71.389 

Coefficients:
                     Estimate Std. Error t value Pr(>|t|)    
EvaporationfactorAH  14.1760     0.2536   55.89   <2e-16 ***
---
Signif. codes:  0 ‘***’ 0.001 ‘**’ 0.01 ‘*’ 0.05 ‘.’ 0.1 ‘ ’ 1

Residual standard error: 25.96 on 209 degrees of freedom
Multiple R-squared:  0.9373,	Adjusted R-squared:  0.937 
F-statistic:  3124 on 1 and 209 DF,  p-value: < 2.2e-16
```

S.9 Air flow

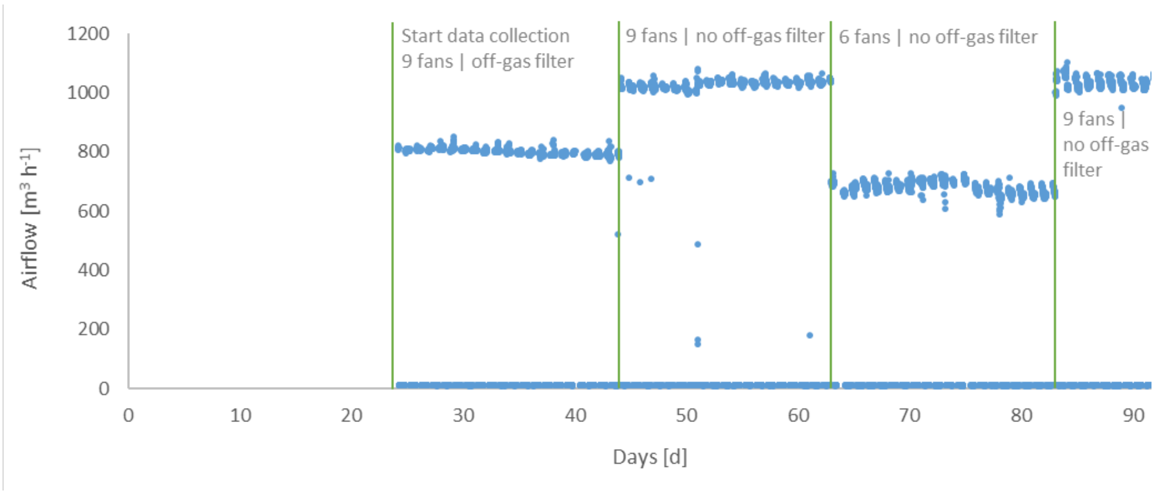

Figure S.11: Air flow measured throughout the field test FT_{Durban} with settings A: 9 fans + off-gas filter, B: 9 fans w/o off-gas filter, C: 6 fans w/o off-gas filter and D: 6 fans + off-gas filter

S.10 Additive cost calculations

Table S.5: Cost calculation of different additives used

Currency exchange:			
	€ -> CHF	1.05226	(16.04.2020)
	ZAR -> CHF	0.0517826	(16.04.2020)
	CHF -> $	1.08	(29.09.2020)
	€ -> $	1.17	(29.09.2020)

Provider	Name	Grade [%]	Size [kg]	Prize [€;ZAR;CHF/bundle]	[$/kg]	Source
Obi hardware store (D, €)	Weiskalkhydrat	93.5%	25	7.99	0.37	https://www.obi.de/zemente-kalke/weisskalkhydrat-25-kg/p/8141798, last access: 16.04.2020
Kalkor (SA)	Hydrated Lime	92.0%	1500	2981.36	0.11	Email request
Vietnam trades	Phosphoric acid	Lab grade	2.5	27.59	11.04	https://www.vietnamtrades.com/vietnam-export-data/phosphoric-acid.html
		85.0%	23940	22254	0.93	https://www.vietnamtrades.com/vietnam-export-data/phosphoric-acid.html
Lab Alley	Phosphoric acid	85.0%	22.7	250	11.01	https://www.laballey.com/collections/phosphoric-acid/products/phosphoric-acid-85-food-grade
		85.0%	1227.44	5460	4.45	https://www.laballey.com/collections/phosphoric-acid/products/phosphoric-acid-85-food-grade
ScienceCompany	Sulphoric acid	95.0 - 97.0%	1	45	45.00	https://www.sciencecompany.com/Sulfuric-Acid-Concentrated-32oz-P6550.aspx
Lab Alley		92-94%	20	140	7.00	https://www.laballey.com/products/sulfuric-acid-lab

Cost calculation Paper	Liters treated per year [L/a]	Additive	Amount additive used [g/L]	[kg/yr]	Specific cost [USD/kg]	Total cost [USD/yr]	Persons [#]	Total cost/ pers [USD/yr pers]	[EUR/yr pers]
FT_Eawag/ Au/ Durban	3650	Ca(OH)2 GER	6	22	0.37	8.19	10	0.82	0.70
	3650	Ca(OH)2 SA	6	22	0.11	2.43	10	0.24	0.21
Mixed ash&lime	3650	Ash	50	183	0	0.00	10	0.00	0.00
	3650		145	529	0	0.00	10	0.00	0.00
	3650	Ca(OH)2 GER	50	183	0.37	68.24	10	6.82	5.83
	3650		145	529	0.37	197.90	10	19.79	16.91
	3650	Ca(OH)2 SA	50	183	0.11	20.29	10	2.03	1.73
	3650		145	529	0.11	58.83	10	5.88	5.03
Acid	3650	Sulphoric acid (96%)	28	101	7.00	709.27	10	70.93	60.62
	3650		28	101	45.00	4559.58	10	455.96	389.71
	3650	Phosphoric acid (89%)	31	115	0.93	106.74	10	10.67	9.12
	3650		31	115	11.01	1264.64	10	126.46	108.09

$Ca(OH)_2$ or hydrated lime is a cheap product used in the building industry and available around the world. Product prices depend on the trading unit, the purity grade and the localization. While in Europe the cost would result in 0.37 USD kg^{-1} (entity: 25 kg, grade: 93.5%) {Obi, 2020 #187}, this would be 0.11 USD kg^{-1} in South Africa (entity: 60 x 25 kg) {Kalkor, 2020 #188}.

S.11 Pathogen testing

Table S.6: Results of pathogen testing at the end of a harvesting period of FT3

**Bacteria**	**E.Coli** [MPN/100 mL]	**Total coliforms** [MPN/100 mL]
Evap.. reactor [4 samples]	**<1**	**<1**

**Helminths**	**Ascaris**				**Ascaris eggs**		**Trichuris**		**Taenia**		**Hook.worm**	
	Infertile	Dead	Necrotic larva	Immotile larva	Undeve-loped.	Deve-loping	Dead	Pot. viable	Dead	Pot. vuable	Undeve-loped.	Deve-loping
Stab. reactor [500 mL sample]	0	0	0	0	0	1	0	0	0	0	0	0
Evap.. reactor [300 mL sample]	0	0	0	0	0	0	0	0	0	0	0	0
